# Incident Lung Cancer and Mortality: Data From Health ABC Using Periodontal Status and Tooth Loss

**DOI:** 10.1111/odi.70178

**Published:** 2026-01-04

**Authors:** Taciane Menezes da Silveira, Eleanor M. Simonsick, Juliana Balbinot Hilgert, Fernando Neves Hugo, Francisco Wilker Mustafa Gomes Muniz, Natália Marcumini Pola

**Affiliations:** ^1^ Graduate Dentistry Program Universidade Federal de Pelotas Pelotas Rio Grande do Sul Brazil; ^2^ Longitudinal Studies Section, Translational Gerontology Branch, National Institute on Aging Intramural Research Program Baltimore Maryland USA; ^3^ Department of Preventive and Social Dentistry Universidade Federal do Rio Grande do Sul Porto Alegre Rio Grande do Sul Brazil; ^4^ Department of Epidemiology & Health Promotion New York University New York New York USA; ^5^ Department of Semiology and Clinic Universidade Federal de Pelotas Pelotas Rio Grande do Sul Brazil

**Keywords:** aging, lung cancer, periodontitis, tooth loss

## Abstract

**Aim:**

To evaluate whether periodontal parameters and tooth loss are associated with lung cancer incidence and mortality among older adults.

**Materials and Methods:**

Data are from the Health, Aging and Body Composition (Health ABC) study, which included 1136 older adults who received a periodontal examination and were followed for up to 16 years. Pocket depth and clinical attachment level were assessed for all present teeth. Adjusted Cox regression models were used to examine the relationship between periodontal parameters and number of natural teeth with lung cancer incidence and mortality.

**Results:**

Lung cancer incidence was 4.4% (*n* = 50) and was identified as cause of death in 3.3% (*n* = 38). Those presenting at least 10% of sites with probing depth ≥ 6 mm demonstrated a higher risk of lung cancer and death due to lung cancer (hazard ratio [HR]: 3.12, 95% confidence interval [CI]: 1.45–6.72 and HR: 3.08, 95% CI: 1.27–7.46), respectively. Tooth loss was associated with a reduced hazard of lung cancer incidence (HR: 0.96; 95% CI: 0.93–0.99) and related mortality (HR: 0.96; 95% CI: 0.92–0.99).

**Conclusion:**

The association between severe periodontal disease parameters and tooth loss with lung cancer among older adults underscores the importance of oral health maintenance for cancer prevention strategies.

## 1 Introduction

Malignant neoplasms are lesions characterized by abnormal, uncontrolled, and autonomous cellular proliferation, generally involving alterations in genes or proteins that regulate cell multiplication and differentiation (IARC 2020). Cancer has a heterogeneous etiology, yet diverse tumor types share common functional markers—known as the hallmarks of cancer—including sustained proliferation, immune evasion, and resistance to cell death. These hallmarks reflect underlying biological processes that, despite tissue‐specific variations, are broadly involved in the multistep development of malignant neoplasms (Hanahan 2022). Worldwide, lung cancer is the second most common malignancy and the leading cause of cancer‐related deaths (Sung et al. 2021). In 2018, the Global Cancer Observatory estimated 2.09 million (11.6%) new cases and 1.76 million (18.4%) deaths from lung cancer globally (Bray et al. 2018).

The association between periodontitis and lung cancer has been reported in several studies (Baima et al. 2024; Mai et al. 2014; Michaud et al. 2017; Verma et al. 2023; Wang et al. 2020; Zeng et al. 2016), with research emphasizing the importance of adjusting for confounders that include age and tobacco consumption (Verma et al. 2023; Wang et al. 2020; Zeng et al. 2016). While both lung cancer and chronic periodontal disease share common risk factors, especially tobacco, the latter has been implicated as an independent risk factor for lung cancer (Chen et al. 2020; Verma et al. 2023; Wang et al. 2020; Zeng et al. 2016). Chronic systemic inflammation from periodontitis may contribute to lung carcinogenesis by inducing reactive oxygen and nitrogen species and activating tumorigenic pathways including NF‐κB (Grivennikov et al. 2010; Lin and Karin 2007). Additionally, inflammatory cytokines including interleukin‐6 (IL‐6), tumoral necrosis factor‐α (TNF‐α), and C‐reactive protein, often elevated in patients with periodontitis, have been associated with increased cancer risk (Kesharani et al. 2022; Pine et al. 2011). Periodontitis can also facilitate bacterial translocation to the lungs via micro‐aspiration, promoting an inflammatory microenvironment that favors tumor progression (Baima et al. 2022, 2024).

Given that lung cancer and periodontitis both affect life quality and longevity of the worldwide population, exploring their potential association may uncover shared biological mechanisms—such as systemic inflammation—that could inform integrated preventive strategies and risk reduction approaches (Demb et al. 2019; Walther et al. 2024). Indeed, a meta‐analysis of cohort studies estimated that periodontal disease was associated with a 24% higher risk of lung cancer (95% CI, 1.13–1.36, *I* ^2^ = 30%) (Zeng et al. 2016). However, important limitations hinder causal inference. According to the GRADE approach, the overall quality of evidence remains low to moderate due to the observational nature of available studies, which are inherently susceptible to residual confounding, reverse causation, and selection bias. Many studies rely on self‐reported periodontal status or partial‐mouth assessments, which may misclassify disease presence and severity (Chen et al. 2020; Trindade et al. 2023). Additionally, study populations often include younger, healthier individuals, which may dilute these associations, making them harder to detect and limiting generalizability to older or more medically complex populations (Ng et al. 2020). Aiming to address these gaps in literature, the present study uses data from the Health, Aging, and Body Composition (Health ABC) Study, an older population with complete periodontal examinations, to test the hypothesis that chronic periodontal disease and number of teeth are associated with lung cancer incidence and mortality.

## 2 Material and Methods

### 2.1 Study Population

This study presents a secondary longitudinal analysis of data from participants enrolled in the Health ABC study, a cohort of participants aged 70–79 years at baseline. This report follows the guidelines of the Strengthening Reporting of Observational Studies in Epidemiology checklist (Knottnerus and Tugwell 2008).

Briefly, a random sample of white Medicare beneficiaries and all black age‐eligible residents were recruited between the years of 1997 and 1998 in Pittsburgh, Pennsylvania, and Memphis, Tennessee. All participants gave written informed consent, and the institutional review boards at both locations approved all study protocols. The study adhered to the principles outlined in the 1975 Declaration of Helsinki. Additional information on the sampling strategy is available in the published literature (Simonsick et al. 2001). The recruitment process involved contacting participants by mail, followed by a phone interview to assess eligibility, and followed by an in‐person visit to the participant's home to confirm eligibility.

Participants were eligible for inclusion if they were well‐functioning, meaning they reported no difficulty walking a quarter mile, climbing 10 steps without resting, or performing basic activities, and did not require the use of an assistive walking device. Additionally, they had to be living in the community and not undergoing active cancer treatment or planning to relocate within the next 3 years. Participants underwent clinical examinations at determined time points and received phone calls every 6 months to report updates on their functional and health status, continuing for up to 16 years. Monitoring specific diseases and recording of deaths were conducted over the 16‐year follow‐up period.

For the present analysis, only individuals with available periodontal data were included. These individuals were then divided into two groups based on lung cancer incidence: (I) those who were diagnosed with lung cancer at any point during the study period, and (II) those who did not experience any lung cancer incidence throughout the study and follow‐up duration. Lung cancer incidence was ascertained through adjudicated medical records, and the date of diagnosis was recorded as the earliest date of confirmed diagnosis documented in the dataset. All diagnoses were verified using standardized criteria or based on ICD codes. Additionally, for lung cancer mortality, individuals were classified into two groups: (I) those for whom lung cancer was the underlying cause of death and (II) those who did not die from lung cancer. The time variable was calculated as the number of days from study enrollment until (1) the date of occurrence of the outcome, (2) the date of the total follow‐up period (16 years), or (3) the date on which the person was censored (stopped being followed) but did not have the outcome.

To evaluate potential selection bias, baseline characteristics of participants included in the periodontal sub‐study were compared with those of the remaining Health ABC cohort members who were either edentulous, systemically unwell, or did not participate in the dental examination. Sociodemographic and health‐related variables were compared using chi‐square or Mann–Whitney tests as appropriate (Table S1).

### 2.2 Oral Health Assessment

The oral health assessment was performed once, either at year two or year three of follow‐up, depending on examiner availability, and included a fullmouth periodontal examination, soft and hard tissue examination, and tooth count. Periodontal evaluation was conducted at six sites per tooth on all teeth, excluding third molars, using a periodontal probe (UNC‐15, Hu‐Friedy, Chicago, IL). Measurements for Pocket Depth (PD) and Clinical Attachment Level (CAL) were recorded by either a trained periodontist or a dental hygienist. A minimum agreement rate of 90% was achieved before starting data collection.

Patient‐centered periodontal variables were recorded in databases and made available for subsequent analyses, as follows: number of sites presenting categorized measures based on predefined cut off points for PD (≥ 4 mm, ≥ 5 mm, and ≥ 6 mm); and number of sites presenting CAL ≥ 3 mm. Recorded values were chosen to represent moderate to deep pockets and moderate clinical attachment loss, which may allow indirect evaluation of periodontal status.

### 2.3 Outcomes Assessment

Lung cancer incidence and mortality were defined as the primary outcomes of the present study. For lung cancer‐related outcome analysis, medical records or death certificate data were accessed. Lung cancer incidence was dichotomized including as cases all individuals meeting at least one of the following criteria: (1) a written pathology report (including cytology or autopsy) confirming malignant neoplasia; (2) a written report from radiologic or other noninvasive tests (computed tomography, magnetic resonance imaging, mammogram, ultrasound, etc.) consistent with malignancy; (3) other written reports by a physician, such as clinic notes, progress notes, operative reports, or discharge summaries indicating the presence of malignancy; or (4) a death certificate listing malignancy as the underlying cause. For lung cancer mortality, the dichotomization was based solely on the presence or absence of criterion (4). For both outcomes, data were collected annually or twice a year (further details can be found at: https://www.nia.nih.gov/sites/default/files/2023‐12/healthabc‐measures‐and‐frequency‐questionnaire.pdf).

### 2.4 Exposure and Covariates

As the available periodontal data were collected only once, the measurements were treated as fixed baseline exposures for all analyses. Different thresholds of periodontal parameters were used to classify the extent and severity of chronic periodontal disease based on PD and CAL.

For PD, participants were categorized into three groups based on the percentage of sites with PD meeting specific thresholds: those with at least 10% of sites having PD ≥ 4 mm, PD ≥ 5 mm, and PD ≥ 6 mm (Armitage 1999; Muniz et al. 2023). For CAL, the number of sites with CAL ≥ 3 mm was considered, as this reflects the history and extent of periodontal disease. Similarly, participants were classified into three categories based on the proportion of affected sites: those with at least 10%, 20%, or 30% of sites exhibiting CAL ≥ 3 mm (Muniz et al. 2023).

Regarding tooth loss, two exposure variables were established: the mean number of natural teeth and the presence of severe tooth loss (yes or no), which was defined as having lost at least 20 teeth (Marcenes et al. 2013).

Sociodemographic and behavioral variables were collected through self‐reported questionnaires. This included sex (male or female), age at baseline (in years), self‐identified race (White or Black), and marital status (single/divorced, married, or widowed). Educational attainment was classified as up to 12th grade or higher education. Smoking status was determined based on the question: “Have you smoked at least 100 cigarettes in your entire life?” with responses categorized as never, former, or current smoker. To account for smoking intensity, we derived pack‐years from baseline data (packs per day × years smoked) and analyzed it both as a continuous variable and using the following categories: 0, ≤ 20, 21–40, and > 40 pack‐years. Sensitivity analyses included models adjusted for smoking status and pack‐years together with alcohol consumption, diabetes, and body mass index (BMI), and models stratified by smoking status.

Alcohol consumption was assessed by asking, “During the past 12 months, how many drinks did you have in a typical week?” and categorized as none/less than one per week, or at least one per week (U.S. Department of Health and Human Services and U.S. Department of Agriculture 2020).

Anthropometric data were obtained during clinic visits conducted by the research team. BMI was calculated as weight (kg) divided by height squared (m^2^), and the mean value was used for analysis. Diabetes status was determined based on a combination of self‐reported physician diagnosis and current use of diabetes‐related medication, categorized as “yes” or “no.”

### 2.5 Statistical Analysis

**FIGURE 1:**
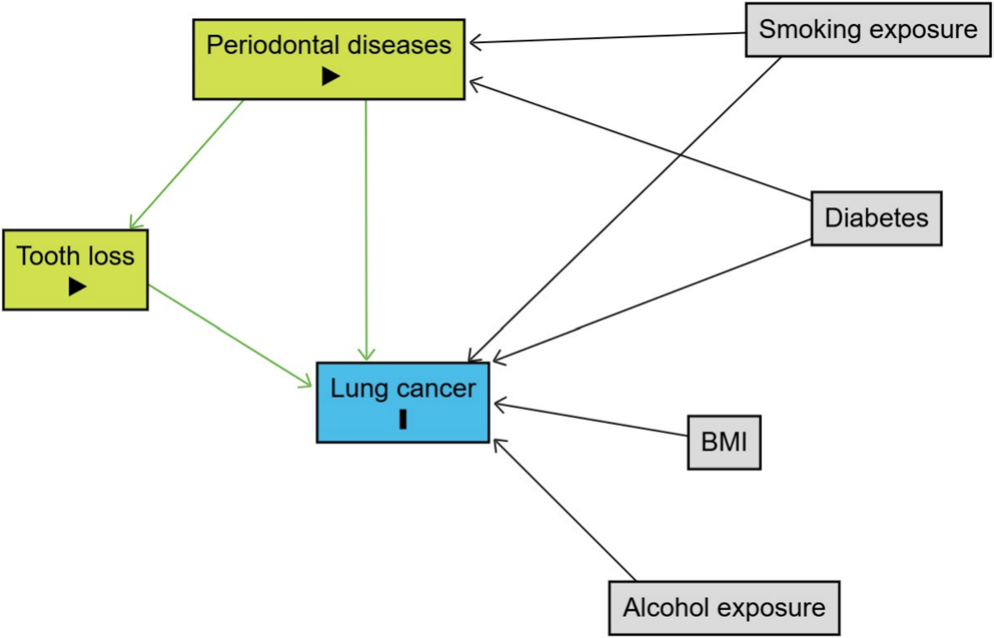
DAG depicting the hypothesized causal pathway between periodontal diseases and lung cancer, adjusted for key confounders. BMI, Body mass index.

The sample was categorized in a dichotomous way for both primary and secondary outcomes. Uni‐ and multivariable analyses were also performed using Cox regression to estimate hazard ratios (HR) and assess the relationship between the covariates and lung cancer incidence and mortality. PD, CAL, and tooth loss were considered the main exposure variables. To identify a minimally sufficient set of covariates for adjustment in the multivariable Cox proportional hazards regression model, we constructed a Directed Acyclic Graph (DAG) using DAGitty (www.dagitty.net) (Figure 1). Covariates identified as potential confounders were included in the regression models established based on theoretical considerations and existing literature. Behavioral factors (smoking exposure and alcohol consumption) were included as influencing both periodontal status and lung cancer. Clinical conditions (BMI and diabetes) were considered as potential confounders given their known associations with both periodontal disease and cancer. This causal framework, supported by previous studies (Bray et al. 2024; Chen et al. 2020; Demb et al. 2019; Goto et al. 2020; Wang et al. 2020; Zeng et al. 2016) guided the selection of confounding variables included in the adjusted models (Merchant and Pitiphat 2002). To ensure reliability of the model, multicollinearity analyses were performed among the independent variables, and none were observed. Independent analyses were performed for each outcome and each primary exposure. As a sensitivity analysis to address potential reverse causality, we ran additional Cox regression models after excluding participants who developed lung cancer within the first 2 years of follow‐up.

The proportional hazards assumption was evaluated for each Cox model using Schoenfeld residuals, and fine–Gray subdistribution hazard models were performed to account for non–lung‐cancer death as a competing risk. All analyses were performed using SPSS, version 21.0 (SPSS, version 21.0, IBM Corp., Armonk, NY, USA) and R Statistical Software, version 4.5.1 (Posit Team 2025). RStudio: Integrated Development Environment for R.

The analyses were performed using complete cases only. Given the small proportion and random distribution of missing values, the potential for bias due to missingness was considered minimal.

## 3 Results

**FIGURE 2:**
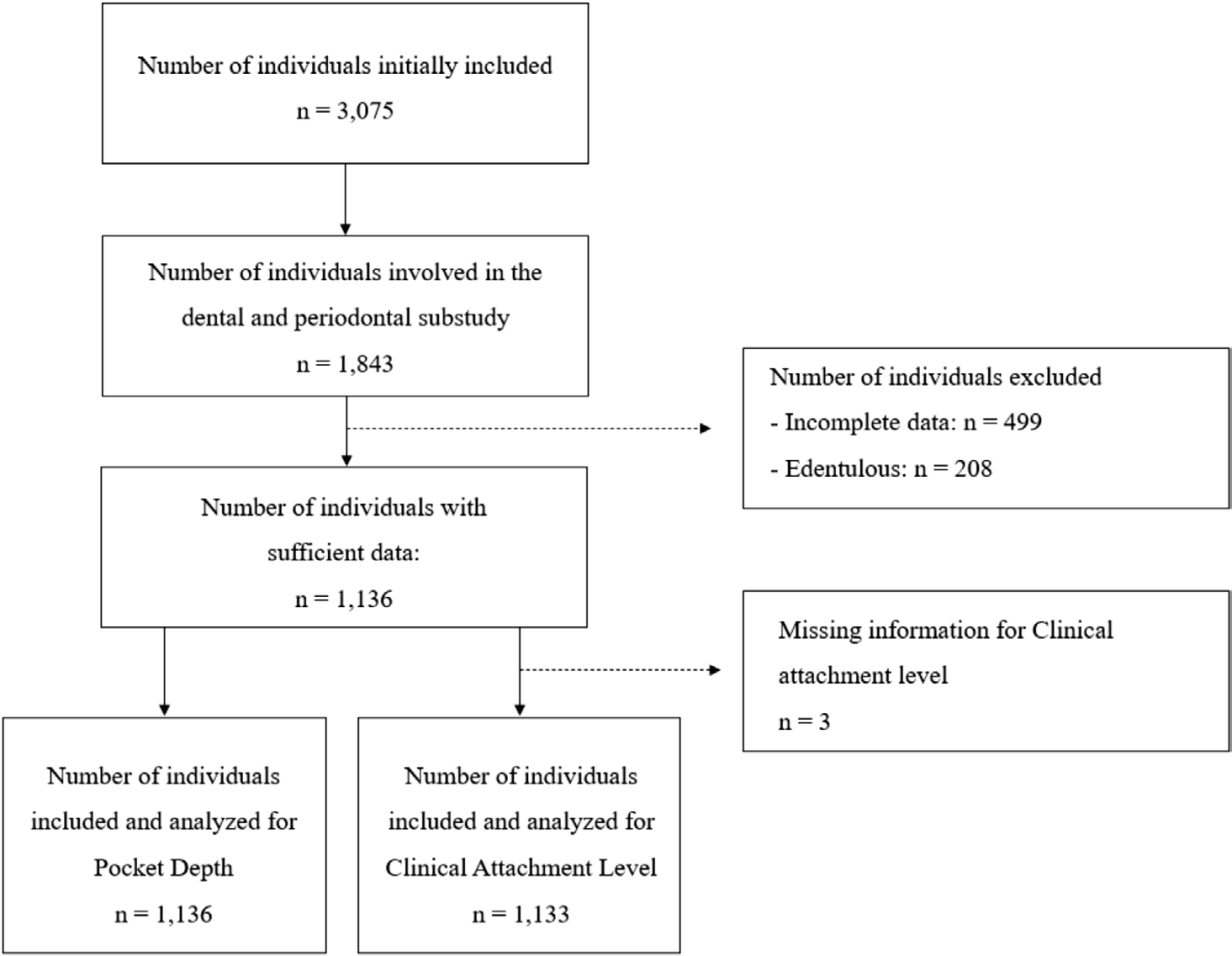
Participants flowchart.

Of the 3075 participants enrolled at baseline, 1843 underwent an oral examination, which was conducted only on days when a periodontal examiner was available. Of these, 38 declined participation, 208 were edentulous, and 441 required prophylactic antibiotics, leading to their exclusion. The final sample included 1136 individuals (36.9% of the sample) for PD analysis, while three participants with insufficient CAL data were excluded from CAL‐related analyses. Figure 2 illustrates the participant selection process. Baseline characteristics of the included and excluded participants are shown in Table S1. The analytic cohort (*n* = 1136) and the excluded individuals (*n* = 1939) were comparable regarding age, sex, and BMI. Participants included in the present analysis were more frequently white, married, and had higher education levels, lower prevalence of diabetes, and slightly lower rates of smoking and alcohol consumption. These differences were expected given the exclusion of edentulous and systemically unwell participants from the dental examination. Results were virtually unchanged when participants who developed lung cancer within the first 2 years of follow‐up were excluded (Tables S2 and S3).

Of 1136 individuals, 566 (49.8%) were men. Table 1 presents the distribution of sociodemographic, medical, behavioral, and dental information according to lung cancer incidence and mortality. In the study population, 4.4% (*n* = 50) were diagnosed with lung cancer, and 3.3% (*n* = 38) died due to lung cancer at some point in the study follow‐up period. The average time to lung cancer incidence was 7.93 ± 4.07 years, while the mean time from the start of follow‐up to lung cancer‐related mortality was 8.98 ± 4.02 years. A smaller number of natural teeth and severe tooth loss were more common among those who developed lung cancer and those who died from it. Moreover, lung cancer incidence and mortality were higher among those who presented at least 10% of sites with PD ≥ 6 mm. Lung cancer incidence and mortality over time according to the assessed periodontal parameters and severe tooth loss are presented in Figures S1–S14.

**TABLE 1:** Baseline characteristics of participants stratified by lung cancer outcomes (*n* = 1136).

	Lung cancer incidence		Mortality due to lung cancer	
	Yes (*n* = 50; 4.4%)	No (*n* = 1086; 95.6%)	Yes (*n* = 38; 3.3%)	No (*n* = 1098; 96.7%)
**Sociodemographic, behavioral, and medical information**				
Sex				
Male—*n* (%)	28 (56.0)	538 (49.5)	21 (55.3)	545 (49.6)
Female—*n* (%)	22 (44.0)	548 (50.5)	17 (44.7)	553 (50.4)
Age (year 1)	73.20 ± 2.58	73.55 ± 2.83	73.18 ± 2.72	73.55 ± 2.83
Mean ± SD (median—IQ)	72.50 (71.00–75.00)	73.00 (71.00–76.00)	72.00 (71.00–75.25)	73.00 (71.00–76.00)
Skin color				
White—*n* (%)	33 (66.0)	700 (64.5)	28 (73.7)	705 (64.2)
Black—*n* (%)	17 (34.0)	386 (35.5)	10 (26.3)	393 (35.8)
Marital status				
Single/divorced—*n* (%)	10 (22.2)	140 (14.0)	7 (20.6)	143 (14.1)
Married—*n* (%)	26 (57.8)	576 (57.5)	21 (61.8)	581 (57.4)
Widow—*n* (%)	9 (20.0)	285 (28.5)	6 (17.6)	288 (28.5)
Missing	5	85	4	86
Schooling				
Up to 12th Grade—*n* (%)	23 (46.0)	474 (43.8)	18 (47.4)	479 (43.8)
Higher—*n* (%)	27 (54.0)	607 (56.2)	20 (52.6)	614 (56.2)
Missing	0	5	0	5
Smoking exposure in 1^st^ year				
Never—*n* (%)	6 (12.0)	529 (48.8)	5 (13.2)	530 (48.4)
Current/former—*n* (%)	44 (88.0)	555 (51.2)	33 (86.8)	566 (51.6)
Missing	0	2	0	2
Smoking (pack‐years)	41.69 (32.97)	16.33 (26.49)	43.74 (33.08)	16.51 (26.55)
Mean ± SD (median—IQ)	34.00 (17.50–55.00)	1.00 (0–26.00)	38.50 (23.25–56.25)	1.00 (0–26.00)
Smoking (pack‐years)				
Zero—*n* (%)	6 (12.2)	529 (49.3)	5 (13.2)	530 (48.9)
≤ 20—*n* (%)	8 (16.3)	239 (22.3)	4 (10.5)	243 (22.4)
21–40—*n* (%)	12 (24.5)	140 (13.1)	10 (26.3)	142 (13.1)
> 40—*n* (%)	23 (46.9)	164 (15.3)	19 (50.0)	168 (15.5)
Alcohol exposure in 1^st^ year				
None or < 1 per week—*n* (%)	28 (56.0)	732 (67.7)	20 (52.6)	740 (67.7)
At least 1 per week—*n* (%)	22 (44.0)	349 (32.3)	18 (47.4)	353 (32.3)
Missing	0	5	0	5
BMI at 1st year	26.43 ± 4.14	27.34 ± 4.68	26.84 ± 4.38	27.32 ± 4.67
Mean ± SD (median—IQ)	26.57 (23.53–28.37)	26.68 (24.20–29.74)	26.66 (23.90–28.48)	26.65 (24.19–29.69)
Diabetes at 1^st^ year				
No—*n* (%)	43 (86.0)	958 (88.2)	34 (89.5)	967 (88.1)
Yes—*n* (%)	7 (5.9)	129 (11.8)	4 (10.5)	131 (11.9)
Missing	0	0	0	0
**Dental characteristics**				
Number of present teeth	16.96 ± 8.39	19.77 ± 7.52	16.39 ± 8.25	19.76 ± 7.53
Mean ± SD (median—IQ)	17.50 (9.00–24.00)	22.00 (15.00–26.00)	16.00 (9.00–24.00)	22.00 (15.00–26.00)
Severe tooth loss				
No—*n* (%)	33 (66.0)	867 (79.8)	24 (63.2)	876 (79.8)
Yes—*n* (%)	17 (34.0)	219 (20.2)	14 (36.8)	222 (20.2)
PD	2.16 ± 0.83	2.06 ± 0.81	2.20 ± 0.80	2.06 ± 0.81
Mean ± SD (median—IQ)	2.11 (1.60–2.58)	1.94 (1.56–2.45)	2.12 (1.65–2.58)	1.94 (1.56–2.45)
At least 10% of sites with PD ≥ 4 mm				
No—*n* (%)	33 (66.0)	724 (66.7)	24 (63.2)	733 (66.8)
Yes—*n* (%)	17 (34.0)	362 (33.3)	14 (36.8)	365 (33.2)
At least 10% of sites with PD ≥ 5 mm				
No—*n* (%)	38 (76.0)	935 (86.1)	29 (76.3)	944 (86.0)
Yes—*n* (%)	12 (24.0)	151 (13.9)	9 (23.7)	154 (14.0)
At least 10% of sites with PD ≥ 6 mm				
No—*n* (%)	42 (84.0)	1019 (93.8)	32 (84.2)	1029 (93.7)
Yes—*n* (%)	8 (16.0)	67 (6.2)	6 (15.8)	69 (6.3)
CAL	2.69 ± 1.91	2.29 ± 1.42	2.84 ± 1.94	2.29 ± 1.42
Mean ± SD (median—IQ)	2.28 (1.25–3.21)	1.86 (1.32–2.88)	2.38 (1.47–3.27)	1.85 (1.32–2.88)
Missing	0	3	0	3
At least 10% of sites with 3 mm CAL				
No—*n* (%)	7 (14.0)	193 (17.8)	4 (10.5)	196 (17.9)
Yes—*n* (%)	43 (86.0)	890 (82.2)	34 (89.5)	899 (82.1)
At least 20% of sites with 3 mm CAL				
No—*n* (%)	16 (32.0)	396 (36.6)	10 (26.3)	402 (36.7)
Yes—*n* (%)	34 (68.0)	687 (63.4)	28 (73.7)	693 (63.3)
At least 30% of sites with 3 mm CAL				
No—*n* (%)	20 (40.0)	567 (52.4)	13 (34.2)	574 (52.4)
Yes—*n* (%)	30 (60.0)	516 (47.6)	25 (65.8)	521 (47.6)

Abbreviations: CAL, clinical attachment level; IQ, interquartile range; PD, Probing depth; SD, standard deviation.

Table S4 shows the univariate regression results that reveal smoking exposure during the first year was significantly associated with both lung cancer incidence (HR: 7.27, 95% CI 3.10–17.06) and mortality (HR: 6.55, 95% CI 2.56–16.79). Additionally, alcohol exposure at least once per week in the first year was significantly associated with increased lung cancer mortality (HR: 1.91, 95% CI 1.01–3.61). Regarding the periodontal parameter CAL, mean values were significantly higher among those who died due to lung cancer (HR: 1.25, 95% CI 1.06–1.49), and the hazard of mortality was 2.11 times higher (95% CI 1.08–4.13) for participants with at least 30% of sites with CAL ≥ 3 mm.

In unadjusted analysis, the number of present teeth was inversely associated with both lung cancer incidence (HR: 0.95, 95% CI: 0.92–0.99) and mortality (HR: 0.95, 95% CI: 0.91–0.98). Table 2 shows the results of the adjusted multivariate analysis, in which the number of teeth remained a protective factor for lung cancer incidence after adjusting for smoking, alcohol exposure, diabetes, and BMI (HR: 0.96, 95% CI: 0.93–0.99). Also, the inverse association between the number of teeth and lung cancer mortality remained statistically significant (HR: 0.95, 95% CI: 0.92–0.99). Individuals with at least 10% of sites with PD ≥ 6 mm had HR of 2.87 (95% CI, 1.34–6.14) for developing lung cancer and 3.00 (95% CI, 1.25–7.19) for lung cancer mortality.

**TABLE 2:** Multivariate Cox regression analysis for lung cancer outcomes.

	Lung cancer incidence HR (95% CI)	*p*	Lung cancer mortality HR (95% CI)	*p*
Number of present teeth	**0.96 (0.93–0.99)**	**0.041**	**0.95 (0.92–0.99)**	**0.019**
Severe tooth loss				
No	Ref.		Ref.	
Yes	1.52 (0.75–3.08)	0.246	1.72 (0.77–3.81)	0.181
PD	1.18 (0.85–1.66)	0.421	1.27 (0.86–1.86)	0.227
At least 10% of sites with PD ≥ 4 mm				
No	Ref.		Ref.	
Yes	1.08 (0.60–1.94)	0.802	1.25 (0.64–2.43)	0.515
At least 10% of sites with PD ≥ 5 mm				
No	Ref.		Ref.	
Yes	1.84 (0.96–3.53)	0.067	1.86 (0.88–3.94)	0.105
At least 10% of sites with PD ≥ 6 mm				
No	**Ref**.		**Ref**.	
Yes	**2.87 (1.34–6.14)**	**0.007**	**3.00 (1.25–7.19)**	**0.014**
CAL	1.11 (0.94–1.32)	0.205	1.19 (0.99–1.42)	0.065
At least 10% of sites with CAL ≥ 3 mm				
No	Ref.		Ref.	
Yes	1.09 (0.48–2.43)	0.843	1.60 (0.56–4.53)	0.380
At least 20% of sites with CAL ≥ 3 mm				
No	Ref.		Ref.	
Yes	1.12 (0.61–2.03)	0.721	1.54 (0.75–3.19)	0.243
At least 30% of sites with CAL ≥ 3 mm				
No	Ref.		Ref.	
Yes	1.40 (0.79–2.47)	0.252	1.84 (0.93–3.61)	0.078

*Note:* Multivariate models were adjusted for smoking, alcohol exposure, diabetes, and BMI. Bold values mean statistically significant results (*p* < 0.05).

Abbreviations: CAL, clinical attachment level; HR (95% CI), Hazard ratio with 95% confidence interval; PD, Probing depth.

When smoking intensity was taken into account, the results were robust. In multivariable models adjusting for both smoking status and pack‐years (Table S5), participants with ≥ 10% of sites with PD ≥ 6 mm had higher risk of lung cancer incidence (HR 2.95, 95% CI 1.37–6.35, *p* = 0.006) and lung cancer mortality (HR 3.03, 95% CI 1.25–7.30, *p* = 0.014) compared with participants without these deep periodontal pockets. In stratified analyses (Table S6), the associations were present among ever‐smokers (incidence HR 3.54, 95% CI 1.56–8.01, *p* = 0.002; mortality HR 3.52, 95% CI 1.39–8.94, *p* = 0.008), whereas estimates in never‐smokers were imprecise.

Examination of Schoenfeld residuals indicated no substantial violations and the proportional hazards assumption was met for all covariates included in the multivariable Cox models (Figures S15–S20). Sensitivity analyses using Fine–Gray subdistribution models, considering non–lung‐cancer death as a competing event, yielded results consistent in both magnitude and direction with those of the Cox regression (Table S7).

To illustrate the clinical magnitude of the associations, cumulative incidence functions for lung cancer were estimated according to periodontal parameters (Figures S21–S23). The estimated 10‐year incidence was approximately 1.2% in participants with severe periodontal parameters and 0.5% in those without, yielding an absolute difference of about 0.7 percentage points. Similar patterns were observed for increasing thresholds of probing depth, attachment loss, and severe tooth loss.

## 4 Discussion

This longitudinal study of older adults found both chronic periodontal disease and tooth loss increased the risk of incident lung cancer and mortality due to lung cancer, even after accounting for well‐established confounders, especially smoking history. Specifically, PD ≥ 6 mm in at least 10% of the sites was significantly associated with lung cancer incidence and mortality.

These findings suggest that it is not past tissue destruction or cumulative history of periodontitis but rather the presence of periodontal inflammation that may play a more relevant role in cancer development and prognosis. PD is a direct marker of ongoing periodontal inflammation, indicating sites with deep pockets where pathogenic biofilms and an exaggerated immune response act (Papapanou et al. 2018). In contrast, CAL reflects previous attachment loss and may not necessarily imply an ongoing inflammatory burden at the time of assessment. This distinction reinforces the potential biological mechanism linking chronic inflammation, rather than past tissue destruction, to cancer outcomes (Walther et al. 2024).

Periodontal pockets with PD ≥ 6 mm normally are at higher levels of inflammation and may be sources of pro‐inflammatory mediators including IL‐6, TNF‐α, and C‐reactive protein, all of which contribute to systemic inflammation and cancer progression (Kesharani et al. 2022). Likewise, findings from another study with Health ABC data indicated a significant association between higher baseline CRP and IL‐6 concentrations and lung cancer risk (Demb et al. 2019). These higher levels of IL‐6 seem to signal the senescence process, as the body's naturally boosted stress response entails the development of cancer (Campisi 2013). Further, IL‐6 appears to be higher in the lungs of smokers experiencing chronic inflammation (Strzelak et al. 2018). In the present analysis, smoking was a significant factor associated with lung cancer incidence and mortality. Since smoking is the major known risk factor for lung cancer, its inclusion in the model validates the robustness of the findings (Ai et al. 2023; Lai et al. 2024; Lee et al. 2012). Some studies also suggest that alcohol consumption may act synergistically with tobacco in the development of cancer, even though the exact mechanism has not been well established (Larsson et al. 2020; Malhotra et al. 2016).

Additionally, the subgingival microbiome of deep periodontal pockets may contribute to the observed association. Pathogens, such as *Porphyromonas gingivalis and Fusobacterium nucleatum*, frequently detected in sites with increased PD, have been implicated in cancer pathogenesis through immune evasion, inflammation modulation, and direct interactions with host cells (Pu et al. 2020). Indeed, there is evidence that *Porphyromonas gingivalis* can be colonized in lung cancer tissues, as the microenvironment of tumorous tissues is more favorable to the survival of this bacterial species than adjacent lung tissues (Liu et al. 2020).

A smaller number of present teeth was associated with a significantly higher risk of lung cancer incidence and mortality. Tooth loss may reflect oral microbiome alterations, potentially fostering a dysbiotic environment that could influence inflammatory systemic disease processes, including carcinogenesis (Michaud et al. 2017). It may also be related to social and behavioral determinants of health, such as poor access to dental care, inadequate nutrition, and lower socioeconomic status, which can influence cancer incidence, progression, and mortality (Schuch et al. 2019; Zou et al. 2022). Furthermore, smoking, a major risk factor for both periodontal disease and lung cancer, likely plays a key role in this association, which impacts access to both dental and medical care. Collectively, these factors may contribute to the observed relationship between fewer teeth and an increased risk of lung cancer incidence and mortality.

Systematic reviews on this topic have reported similar findings (Chen et al. 2020; Verma et al. 2023; Wang et al. 2020; Zeng et al. 2016), reinforcing the potential association between periodontitis and lung cancer. Regardless of some degree of publication bias, a recent meta‐analysis showed that individuals with periodontitis present a 41% higher risk ratio for lung neoplasm compared with periodontally healthy individuals (95% CI: 1.32–1.52) (Verma et al. 2023). Along with the current results, the association between PD and a significant risk of lung cancer was found both in a meta‐analysis of cohort studies (HR: 1.40, 95% CI: 1.25–1.58; *I* ^2^: 8.7%) and case–control studies (odds ratio [OR]: 1.51, 95% CI: 1.16–1.98; *I* ^2^: 36.5%) (Wang et al. 2020). However, those studies recommend further research with robust methodologies to clarify this relationship and strengthen the existing evidence.

Limitations in the present study must be considered for a clear interpretation of our findings. The Health ABC cohort consists exclusively of older adults aged 70–79 years at baseline. As such, the generalizability of our findings to middle‐aged or younger populations is limited. The biological mechanisms linking periodontal disease and lung cancer may differ across age groups due to variations in the immune system, cumulative exposure to risk factors, and competing health conditions. Therefore, while our results provide important insights into risk stratification and preventive strategies among older adults, caution should be exercised when extrapolating these findings to younger individuals. It's also important to note that the available data did not allow for a definitive case definition of periodontitis. Nonetheless, higher PD thresholds may reflect higher complexity in periodontal treatment needs, as indicated by the Community Periodontal Index of Treatment Needs (CPITN), a widely applied tool in periodontal epidemiology. Similarly, a greater extent of CAL may suggest a higher burden of periodontal diseases, supporting the exploratory approach adopted in this study. In this context, the percentages were defined to represent the extension of periodontal disease, as localized and generalized (Papapanou et al. 2018). However, the absence of data on bleeding on probing, which is the primary clinical marker of periodontal disease activity, may have influenced our findings due to the inability to determine disease activity status in the evaluated population.

A potential confounder in the periodontal disease–lung cancer relationship is smoking intensity. We addressed this by including pack‐years in sensitivity analyses; effect estimates remained essentially unchanged, suggesting residual confounding by smoking is unlikely to fully explain the observed associations. Nonetheless, due to possible measurement error in smoking history and limited power among never‐smokers, we cannot definitively exclude residual confounding. Another limitation of this study is the lack of information on lung cancer treatment, which is a key determinant of survival. Without this data, residual confounding may remain in the mortality analyses, and our findings should be interpreted as reflecting the overall association between baseline periodontal status and lung cancer mortality, without distinguishing effects that could be mediated or modified by treatment. In addition, both exposure variables and covariates were collected only at the beginning of follow‐up. Thus, changes in habits, behaviors, or medical conditions during the 17–18 years of observation could not be captured and may have influenced the results. Information on periodontal treatment during follow‐up was also unavailable, which may have led to unmeasured changes in periodontal status (Güven et al. 2019). Despite these limitations, the study provides valuable evidence from a large, well‐characterized cohort, highlighting the importance of periodontal health in the context of chronic diseases, including cancer.

Overall, our findings indicate that severe periodontal parameters and a smaller number of teeth are associated with greater hazards of lung cancer incidence and associated mortality. These associations may be driven by chronic systemic inflammation and lifestyle factors such as smoking and alcohol consumption. Maintaining periodontal health could be an important consideration in cancer prevention and management. Further research is needed to explore underlying mechanisms and potential clinical implications.

## Author Contributions

**Taciane Menezes da Silveira:** methodology, writing – original draft, conceptualization, formal analysis. **Eleanor M. Simonsick:** funding acquisition, writing – review and editing, investigation, resources. **Juliana Balbinot Hilgert:** validation, visualization, writing – review and editing. **Fernando Neves Hugo:** validation, writing – review and editing, methodology. **Francisco Wilker Mustafa Gomes Muniz:** writing – review and editing, project administration, data curation, supervision, conceptualization. **Natália Marcumini Pola:** writing – review and editing, project administration, supervision, conceptualization.

## Funding

This work was supported by the National Institutes of Health, National Institute of Nursing Research, National Institute on Aging (N01AG62101, N01AG62103, N01AG62106, R01AG028050), Coordenação de Aperfeiçoamento de Pessoal de Nível Superior, Conselho Nacional de Desenvolvimento Científico e Tecnológico, U.S. Department of Health and Human Services.

## Conflicts of Interest

The authors declare no conflicts of interest.

## Supporting information


**Table S1:** Frequency distribution for the older adults included and excluded in the periodontal sub‐study, along with comparison between these individuals. Excluded participants were those who were edentulous, systemically unwell, or not enrolled in the dental and periodontal examination.
**Table S2:** Multivariate Cox regression analysis for lung cancer outcomes excluding participants who developed lung cancer within the first 2 years of follow‐up (*n* = 2).
**Table S3:** Multivariate Cox regression analysis for lung cancer outcomes excluding participants who developed lung cancer within the first 2 years of follow‐up (*n* = 2).
**Table S4:** Univariate Cox regression analysis for lung cancer outcomes.
**Table S5:** Multivariate Cox regression analysis for lung cancer outcomes.
**Table S6:** Multivariate cox regression analysis for lung cancer outcomes stratified by smoking status.
**Table S7:** Fine–Gray subdistribution hazard models accounting for non–lung‐cancer death as a competing event.
**Figure S1:** Lung cancer incidence over time in individuals with and without severe tooth loss.
**Figure S2:** Lung cancer incidence over time in individuals with and without at least 10% of sites with PD ≥ 4 mm.
**Figure S3:** Lung cancer incidence over time in individuals with and without at least 10% of sites with PD ≥ 5 mm.
**Figure S4:** Lung cancer incidence over time in individuals with and without at least 10% of sites with PD ≥ 6 mm.
**Figure S5:** Lung cancer incidence over time in individuals with and without at least 10% of sites with CAL ≥ 3 mm.
**Figure S6:** Lung cancer incidence over time in individuals with and without at least 20% of sites with CAL ≥ 3 mm.
**Figure S7:** Lung cancer incidence over time in individuals with and without at least 30% of sites with CAL ≥ 3 mm.
**Figure S8:** Lung cancer mortality over time in individuals with and without severe tooth loss.
**Figure S9:** Lung cancer mortality over time in individuals with and without at least 10% of sites with PD ≥ 4 mm.
**Figure S10:** Lung cancer mortality over time in individuals with and without at least 10% of sites with PD ≥ 5 mm.
**Figure S11:** Lung cancer mortality over time in individuals with and without at least 10% of sites with PD ≥ 6 mm.
**Figure S12:** Lung cancer mortality over time in individuals with and without at least 10% of sites with CAL ≥ 3 mm.
**Figure S13:** Lung cancer mortality over time in individuals with and without at least 20% of sites with CAL ≥ 3 mm.
**Figure S14:** Lung cancer mortality over time in individuals with and without at least 30% of sites with CAL ≥ 3 mm.
**Figure S15:** Schoenfeld residuals for tooth loss and lung cancer incidence (fully adjusted model).
**Figure S16:** Schoenfeld residuals for Mean PD and lung cancer incidence (fully adjusted model).
**Figure S17:** Schoenfeld residuals for Mean AL and lung cancer incidence (fully adjusted model).
**Figure S18:** Schoenfeld residuals for tooth loss and lung cancer death (fully adjusted model).
**Figure S19:** Schoenfeld residuals for Mean PD and lung cancer death (fully adjusted model).
**Figure S20:** Schoenfeld residuals for Mean AL and lung cancer death (fully adjusted model).
**Figure S21:** Cumulative incidence of lung cancer according to the presence of severe tooth loss.
**Figure S22:** Cumulative incidence of lung cancer by probing depth thresholds (≥ 10% of sites with PD ≥ 4 mm, PD ≥ 5 mm, PD ≥ 6 mm).
**Figure S23:** Cumulative incidence of lung cancer by clinical attachment loss thresholds (≥ 10%, ≥ 20%, ≥ 30% of sites with CAL ≥ 3 mm).

## Data Availability

The data that support the findings of this study are available from the corresponding author upon reasonable request.
