# Supplementary material for: Incident Lung Cancer and Mortality: Data From Health ABC Using Periodontal Status and Tooth Loss
Source: Oral Dis. 2026 Jan 4;32(5):1460–70. doi: 10.1111/odi.70178 (PMC13365001; doi:10.1111/odi.70178)

**SUPPLEMENTAL MATERIAL**

| Table S1. Frequency distribution for the older adults included and excluded in the periodontal sub-study, along with comparison between these individuals. Excluded participants were those who were edentulous, systemically unwell, or not enrolled in the dental and periodontal examination. | | | |
| --- | --- | --- | --- |
|  | **Included participants (n=1136; 36.9%)** | **Excluded participants**  **(n=1939; 63.1%)** | **P-value** |
| Sex  *Male – n (%)*  *Female – n (%)* | 566 (49.8)  570 (50.2) | 925 (47.7)  1014 (52.3) | 0.256* |
| Age (year 1)  *Mean±SD (median – IQ)* | 73.54±2.82  73.00 (71.00 – 76.00) | 73.68±2.90  73.00 (71.00 – 76.00) | 0.216& |
| Skin color  *White – n (%)*  *Non-white – n (%)* | 733 (64.5)  403 (35.5) | 1061 (54.7)  878 (45.3) | 0.000* |
| Marital status  *Single – n (%)*  *Married – n (%)*  *Widow – n (%)*  *Divorced – n (%)*  *Missing* | 58 (5.5)  603 (57.6)  293 (28.0)  92 (8.8)  90 | 86 (4.7)  970 (52.9)  597 (32.6)  181 (9.9)  105 | 0.029* |
| Schooling  *Up to 8^th^ Grade – n (%)*  *Grade 9^th^ to 12^th^ – n (%)*  *Higher – n (%)*  *Missing* | 77 (6.8)  420 (37.1)  634 (56.1)  5 | 246 (12.7)  835 (43.1)  855 (44.2)  3 | <0.001* |
| Smoking exposure at 1^st^ year  *Never – n (%)*  *Current/Former – n (%)*  *Missing* | 535 (47.2)  599 (52.8) | 813 (42.0)  1123 (58.0) | 0.005* |
| Alcohol exposure at 1^st^ year  *None or <1 per week – n (%)*  *At least 1 per week – n (%)*  *Missing* | 760 (67.2)  371 (32.8) | 1421 (73.6)  511 (26.4) | 0.000* |
| BMI at 1^st^ year  *Mean±SD (median – IQ)* | 27.30 (4.65)  26.69 (24.19 – 29.68) | 27.45 (4.92)  27.01 (24.06 – 30.28) | 0.244& |
| Diabetes at 1^st^ year  *No – n (%)*  *Yes – n (%)*  *Missing* | 1004 (88.4)  132 (11.6)  0 | 1608 (83.0)  328 (16.9)  1 | <0.001* |
| Legend: *Chi-square; &Mann-Whitney. IQ: interquartile range. | | | |

| Table S2. Multivariate Cox regression analysis for lung cancer outcomes excluding participants who developed lung cancer within the first two years of follow-up (n = 2). | | | | | | | | |  |
| --- | --- | --- | --- | --- | --- | --- | --- | --- | --- |
|  | **Lung cancer incidence**  **HR (95%CI)** | | **P-value** | | **Lung cancer mortality**  **HR (95%CI)** | | **P-value** | |  |
| Number of present teeth | **0.96 (0.93 – 0.99)** | | **0.048** | | **0.95 (0.92 – 0.99)** | | **0.019** | |  |
| Severe tooth loss  *No*  *Yes* | Ref.  1.41 (0.67 – 2.96) | | 0.357 | | Ref.  1.72 (0.77 – 3.81) | | 0.181 | |  |
| PD | 1.23 (0.86 – 1.74) | | 0.243 | | 1.27 (0.86 – 1.86) | | 0.227 | |  |
| At least 10% of sites with PD ≥4mm  *No*  *Yes* | Ref.  1.15 (0.63 – 2.09) | | 0.637 | | Ref.  1.25 (0.64 – 2.43) | | 0.515 | |  |
| At least 10% of sites with PD ≥5mm  *No*  *Yes* | **Ref.**  **1.96 (1.02 – 3.78)** | | **0.044** | | Ref.  1.86 (0.88 – 3.94) | | 0.105 | |  |
| At least 10% of sites with PD ≥6mm  *No*  *Yes* | **Ref.**  **3.07 (1.42 – 6.59)** | | **0.004** | | **Ref.**  **3.00 (1.25 – 7.19)** | | **0.014** | |  |
| CAL | 1.13 (0.96 – 1.34) | | 0.130 | | 1.19 (0.99 – 1.42) | | 0.065 | |  |
| At least 10% of sites with CAL ≥3mm  *No*  *Yes* | Ref.  1.24 (0.52 – 2.95) | | 0.619 | | Ref.  1.60 (0.56 – 4.53) | | 0.380 | |  |
| At least 20% of sites with CAL ≥3mm  *No*  *Yes* | Ref.  1.16 (0.63 – 2.16) | | 0.620 | | Ref.  1.54 (0.75 – 3.19) | | 0.243 | |  |
| At least 30% of sites with CAL ≥3mm  *No*  *Yes* | Ref.  1.43 (0.80 – 2.57) | | 0.226 | | Ref.  1.84 (0.93 – 3.61) | | 0.078 | |  |
| Legend: CAL: clinical attachment level; PD: Probing depth; HR (95% CI): Hazard ratio with 95% confidence interval; multivariate models were adjusted for smoking, alcohol exposure, diabetes, and BMI. | | | | | | | | |  |
| Table S3. Multivariate Cox regression analysis for lung cancer outcomes excluding participants who developed lung cancer within the first two years of follow-up (n = 2). | | | | | | | | | |
|  | | **Lung cancer incidence**  **HR (95%CI)** | | **P-value** | | **Lung cancer mortality**  **HR (95%CI)** | | **P-value** | |
| Number of present teeth | | 0.96 (0.93 – 1.05) | | 0.094 | | **0.96 (0.92 – 0.99)** | | **0.039** | |
| Severe tooth loss  *No*  *Yes* | | Ref.  1.39 (0.66 – 2.91) | | 0.378 | | Ref.  1.70 (0.77 – 3.77) | | 0.190 | |
| PD | | 1.23 (0.87 – 1.74) | | 0.235 | | 1.27 (0.86 – 1.88) | | 0.213 | |
| At least 10% of sites with PD ≥4mm  *No*  *Yes* | | Ref.  1.17 (0.64 – 2.13) | | 0.602 | | Ref.  1.29 (0.66 – 2.51) | | 0.461 | |
| At least 10% of sites with PD ≥5mm  *No*  *Yes* | | **Ref.**  **1.98 (1.02 – 3.83)** | | **0.041** | | Ref.  1.87 (0.88 – 3.96) | | 0.103 | |
| At least 10% of sites with PD ≥6mm  *No*  *Yes* | | **Ref.**  **3.08 (1.43 – 6.64)** | | **0.004** | | **Ref.**  **3.03 (1.25 – 7.30)** | | **0.014** | |
| CAL | | 1.13 (0.95 – 1.34) | | 0.154 | | 1.18 (0.98 – 1.42) | | 0.078 | |
| At least 10% of sites with CAL ≥3mm  *No*  *Yes* | | Ref.  1.23 (0.51 – 2.93) | | 0.637 | | Ref.  1.57 (0.55 – 4.50) | | 0.399 | |
| At least 20% of sites with CAL ≥3mm  *No*  *Yes* | | Ref.  1.11 (0.60 – 2.06) | | 0.729 | | Ref.  1.45 (0.70 – 3.01) | | 0.316 | |
| At least 30% of sites with CAL ≥3mm  *No*  *Yes* | | Ref.  1.37 (0.76 – 2.46) | | 0.289 | | Ref.  1.74 (0.88 – 3.43) | | 0.110 | |
| Legend: CAL: clinical attachment level; PD: Probing depth; HR (95% CI): Hazard ratio with 95% confidence interval; multivariate models were adjusted for smoking, pack years, alcohol exposure, diabetes, and BMI. | | | | | | | | | |

| Supplementary table S4. Univariate Cox regression analysis for lung cancer outcomes. | | | | |
| --- | --- | --- | --- | --- |
|  | **Lung cancer incidence**  **HR (95%CI)** | **P-value** | **Lung cancer mortality**  **HR (95%CI)** | **P-value** |
| Sociodemographic, behavioral, and medical information | | | | |
| Sex  *Male*  *Female* | Ref.  0.71 (0.41 – 1.24) | 0.229 | Ref.  0.72 (0.38 – 1.36) | 0.312 |
| Age (year 1) | 0.99 (0.89 – 1.09) | 0.785 | 0.99 (0.88 – 1.11) | 0.870 |
| Skin color  *White*  *Black* | Ref.  0.96 (0.54 – 1.73) | 0.900 | Ref.  0.67 (0.32 – 1.37) | 0.270 |
| Marital status  *Single/Divorced*  *Married*  *Widow* | Ref.  0.61 (0.29 – 1.26)  0.43 (0.18 – 1.05) | 0.179  0.063 | Ref.   - 1. (0.29 – 1.63)   0.40 (0.13 – 1.19) | 0.398  0.098 |
| Schooling  *Up to 12^th^ Grade*  *Higher* | Ref.  0.92 (0.53 – 1.61) | 0.773 | Ref.  0.87 (0.46 – 1.65) | 0.679 |
| Smoking exposure in 1^st^ year  *Never*  *Current/Former* | **Ref.**  **7.27 (3.10 – 17.06)** | **<0.001** | **Ref.**  **6.55 (2.56 – 16.79)** | **<0.001** |
| Alcohol exposure in 1^st^ year  *None or <1 per week*  *At least 1 per week* | Ref.  1.67 (0.96 – 2.91) | 0.073 | **Ref.**  **1.91 (1.01 – 3.61)** | **0.047** |
| BMI at 1^st^ year | 0.95 (0.89 – 1.01) | 0.111 | 0.97 (0.90 – 1.04) | 0.394 |
| Diabetes at 1^st^ year  *No*  *Yes* | Ref.  1.40 (0.63 – 3.12) | 0.406 | Ref.  1.04 (0.37 – 2.93) | 0.944 |
| Dental characteristics | | | | |
| Number of present teeth | **0.95 (0.92 – 0.99)** | **0.007** | **0.95 (0.91 – 0.98)** | **0.005** |
| Severe tooth loss  *No*  *Yes* | Ref.  1.91 (0.96 – 3.83) | 0.067 | Ref.  2.05 (0.94 – 4.48) | 0.071 |
| Mean PD | 1.19 (0.87 – 1.62) | 0.272 | 1.25 (0.89 – 1.75) | 0.194 |
| At least 10% of sites with PD ≥4mm  *No*  *Yes* | Ref.  1.10 (0.61 – 1.97) | 0.759 | Ref.  1.26 (0.65 – 2.44) | 0.490 |
| At least 10% of sites with PD ≥5mm  *No*  *Yes* | **Ref.**  **2.06 (1.08 – 3.95)** | **0.029** | Ref.  2.05 (0.97 – 4.34) | 0.059 |
| At least 10% of sites with PD ≥6mm  *No*  *Yes* | **Ref.**  **3.11 (1.46 – 6.63)** | **0.003** | **Ref.**  **3.10 (1.29 – 7.41)** | **0.011** |
| Mean CAL | **1.19 (1.02 – 1.40)** | **0.031** | **1.25 (****1.06 – 1.49)** | **0.010** |
| At least 10% of sites with CAL ≥3mm  *No*  *Yes* | Ref.  1.42 (0.64 – 3.15) | 0.395 | Ref.  1.99 (0.71 – 5.62) | 0.192 |
| At least 20% of sites with CAL ≥3mm  *No*  *Yes* | Ref.  1.28 (0.71 – 2.32) | 0.413 | Ref.  1.71 (0.83 – 3.52) | 0.145 |
| At least 30% of sites with CAL ≥3mm  *No*  *Yes* | Ref.  1.64 (0.93 – 2.89) | 0.086 | **Ref.**  **2.11 (1.08 – 4.13)** | **0.029** |
| Legend: CAL: clinical attachment level; PD: Probing depth; HR (95% CI): Hazard ratio with 95% confidence interval. | | | | |

| Supplementary table S5. Multivariate Cox regression analysis for lung cancer outcomes. | | | | |
| --- | --- | --- | --- | --- |
|  | **Lung cancer incidence**  **HR (95%CI)** | **P-value** | **Lung cancer mortality**  **HR (95%CI)** | **P-value** |
| Number of present teeth | **0.97 (0.93 – 1.00)** | **0.058** | **0.96 (0.92 – 0.99)** | **0.039** |
| Severe tooth loss  *No*  *Yes* | Ref.  1.54 (0.75 – 3.13) | 0.231 | Ref.  1.70 (0.77 – 3.77) | 0.190 |
| PD | 1.20 (0.85 – 1.69) | 0.313 | 1.27 (0.86 – 1.88) | 0.213 |
| At least 10% of sites with PD ≥4mm  *No*  *Yes* | Ref.  1.13 (0.63 – 2.05) | 0.683 | Ref.  1.29 (0.66 – 2.51) | 0.461 |
| At least 10% of sites with PD ≥5mm  *No*  *Yes* | Ref.  1.91 (0.99 – 3.69) | 0.052 | Ref.  1.87 (0.88 – 3.96) | 0.103 |
| At least 10% of sites with PD ≥6mm  *No*  *Yes* | **Ref.**  **2.95 (1.37 – 6.35)** | **0.006** | **Ref.**  **3.03 (1.25 – 7.30)** | **0.014** |
| CAL | 1.12 (0.95 – 1.33) | 0.182 | 1.18 (0.98 – 1.42) | 0.078 |
| At least 10% of sites with CAL ≥3mm  *No*  *Yes* | Ref.  1.27 (0.53 – 3.02) | 0.590 | Ref.  1.57 (0.55 – 4.50) | 0.399 |
| At least 20% of sites with CAL ≥3mm  *No*  *Yes* | Ref.  1.15 (0.61 – 2.12) | 0.662 | Ref.  1.45 (0.70 – 3.01) | 0.316 |
| At least 30% of sites with CAL ≥3mm  *No*  *Yes* | Ref.  1.42 (0.79 – 2.54) | 0.237 | Ref.  1.74 (0.88 – 3.43) | 0.110 |
| Legend: CAL: clinical attachment level; PD: Probing depth; HR (95% CI): Hazard ratio with 95% confidence interval; multivariate models were adjusted for smoking, pack-years, alcohol exposure, diabetes, and BMI. | | | | |

| Supplementary table S6. Multivariate cox regression analysis for lung cancer outcomes stratified by smoking status. | | | | | | | | |
| --- | --- | --- | --- | --- | --- | --- | --- | --- |
|  | **NO** | | | | **YES** | | | |
|  | **Lung cancer incidence**  **HR (95%CI)** | **P-value** | **Lung cancer mortality**  **HR (95%CI)** | **P-value** | **Lung cancer incidence**  **HR (95%CI)** | **P-value** | **Lung cancer mortality**  **HR (95%CI)** | **P-value** |
| Number of present teeth | 1.02 (0.90 – 1.17) | 0.722 | 1.00 (0.87 – 1.14) | 0.997 | **0.96 (0.92 – 0.99)** | **0.025** | **0.95 (0.90 – 0.99)** | **0.016** |
| Severe tooth loss  *no*  *yes* | Collinearity |  | Collinearity |  | Ref.  1.65 (0.77 – 3.51) | 0.197 | Ref.  1.82 (0.77 – 4.32) | 0.174 |
| Pd | 1.12 (0.51 – 2.45) | 0.776 | 1.25 (0.64 – 2.41) | 0.507 | 1.18 (0.79 – 1.76) | 0.424 | 1.23 (0.76 – 1.96) | 0.376 |
| At least 10% of sites with pd ≥4mm  *no*  *yes* | Ref.  1.20 (0.21 – 6.73) | 0.834 | Ref.  1.72 (0.28 – 10.73) | 0.560 | Ref.  0.92 (0.46 – 1.81) | 0.802 | Ref.  0.95 (0.44 – 2.09) | 0.903 |
| At least 10% of sites with pd ≥5mm  *no*  *yes* | Ref.  2.10 (0.23 – 19.45) | 0.513 | Ref.  2.95 (0.30 – 29.39) | 0.357 | Ref.  1.75 (0.84 – 3.67) | 0.137 | Ref.  1.56 (0.65 – 3.71) | 0.320 |
| At least 10% of sites with pd ≥6mm  *no*  *yes* | Collinearity |  | Collinearity |  | **Ref.**  **3.54 (1.56 – 8.01)** | **0.002** | **Ref.**  **3.52 (1.39 – 8.94)** | **0.008** |
| Cal | 0.60 (0.24 – 1.55) | 0.296 | 0.72 (0.27 – 1.91) | 0.508 | 1.13 (0.94 – 1.35) | 0.187 | 1.19 (0.98 – 1.45) | 0.086 |
| At least 10% of sites with cal ≥3mm  *no*  *yes* | Ref.  0.91 (0.22 – 16.74) | 0.558 | Ref.  1.72 (0.19 – 16.02) | 0.633 | Ref.  0.84 (0.35 – 2.03) | 0.697 | Ref.  1.34 (0.40 – 4.49) | 0.639 |
| At least 20% of sites with cal ≥3mm  *no*  *yes* | Ref.  0.79 (0.16 – 3.99) | 0.776 | Ref.  1.25 (0.20 – 7.71) | 0.808 | Ref.  1.09 (0.55 – 2.18) | 0.804 | Ref.  1.53 (0.65 – 3.63) | 0.336 |
| At least 30% of sites with cal ≥3mm  *no*  *yes* | Ref.  0.71 (0.13 – 3.92) | 0.691 | Ref.  0.99 (0.16 – 6.13) | 0.998 | Ref.  1.34 (0.70 – 2.58) | 0.381 | Ref.  1.82 (0.82 – 4.04) | 0.141 |
| Legend: cal: clinical attachment level; pd: probing depth; hr (95% ci): hazard ratio with 95% confidence interval; multivariate models were adjusted for smoking and alcohol exposure, diabetes, bmi, educational level, and marital status. | | | | | | | | |

| Table S7. Fine–Gray subdistribution hazard models accounting for non–lung-cancer death as a competing event. | | | | |
| --- | --- | --- | --- | --- |
|  | **Lung cancer incidence**  **sHR (95%CI)** | **P-value** | **Lung cancer mortality**  **sHR (95%CI)** | **P-value** |
| Number of present teeth | 0.96 (0.93-0.99) | 0.004 | 0.96 (0.93-0.998) | 0.037 |
| Severe tooth loss  *No*  *Yes* | Ref.  1.52 (0.74-3.13) | 0.26 | Ref.  1.60 (0.78-3.30) | 0.20 |
| PD | 1.18 (0.83-1.67) | 0.36 | 1.19 (0.84-1.67) | 0.33 |
| At least 10% of sites with PD ≥4mm  *No*  *Yes* | Ref.  1.08 (0.60-1.94) | 0.80 | Ref.  1.11 (0.62-1.99) | 0.73 |
| At least 10% of sites with PD ≥5mm  *No*  *Yes* | Ref.  1.84 (0.97-3.50) | 0.064 | Ref.  1.90 (1.00-3.61) | 0.049 |
| At least 10% of sites with PD ≥6mm  *No*  *Yes* | Ref.  2.86 (1.36-6.05) | 0.004 | Ref.  3.00 (1.43-6.30) | 0.004 |
| CAL | 1.11 (0.93-1.34) | 0.24 | 1.12 (0.93-1.35) | 0.23 |
| At least 10% of sites with CAL ≥3mm  *No*  *Yes* | Ref.  1.09 (0.49-2.42) | 0.84 | Ref.  1.12 (0.51-2.47) | 0.78 |
| At least 20% of sites with CAL ≥3mm  *No*  *Yes* | Ref.  1.12 (0.62-2.01) | 0.38 | Ref.  1.14 (0.64-2.03) | 0.67 |
| At least 30% of sites with CAL ≥3mm  *No*  *Yes* | Ref.  1.40 (0.80-2.44) | 0.39 | Ref.  1.39 (0.80-2.43) | 0.24 |
| Legend: CAL: clinical attachment level; PD: Probing depth; sHR (95% CI): subdistribution Hazard ratio with 95% confidence interval. | | | | |

**Supplementary Figure S1.** Lung cancer incidence over time in individuals with and without severe tooth loss.


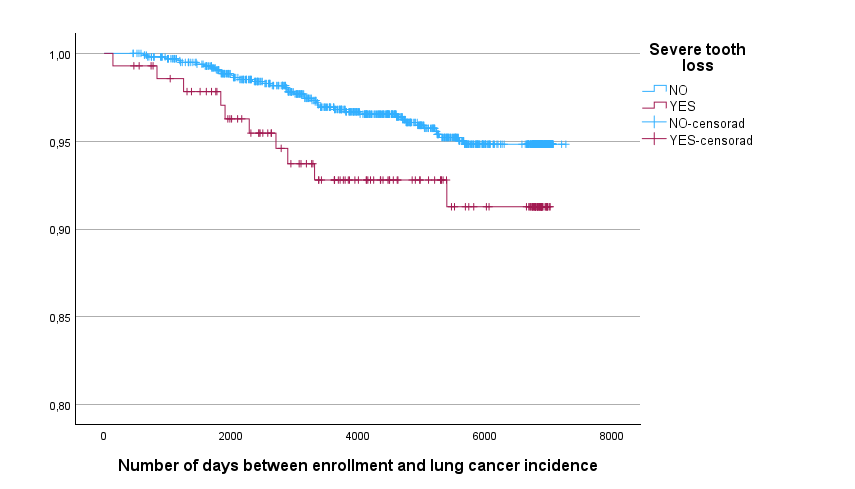


**Supplementary Figure S2.** Lung cancer incidence over time in individuals with and without at least 10% of sites with PD ≥4mm.


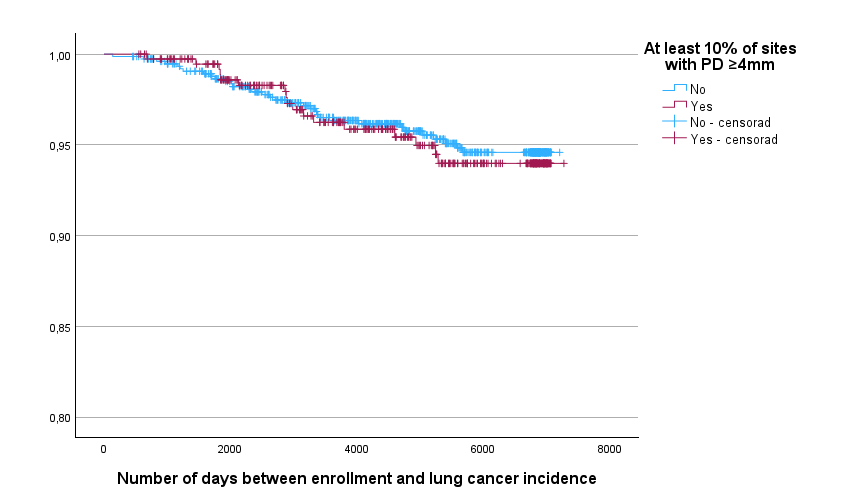


**Supplementary Figure S3**. Lung cancer incidence over time in individuals with and without at least 10% of sites with PD ≥5mm.


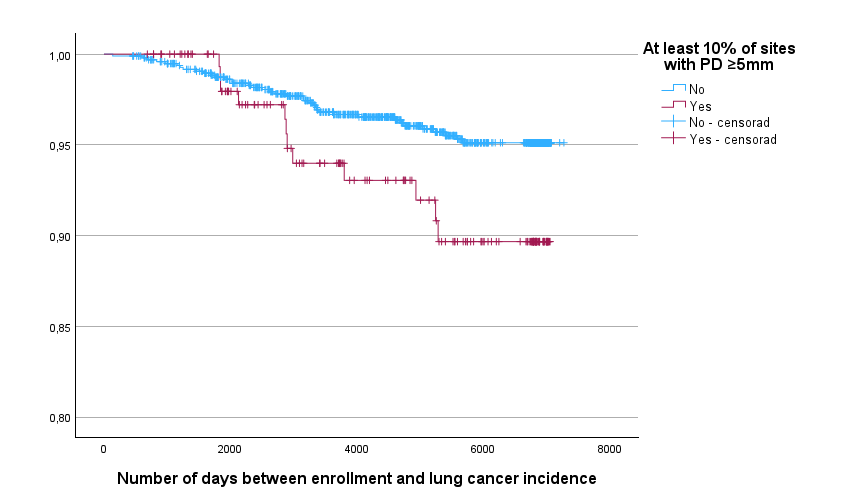


**Supplementary Figure S4.** Lung cancer incidence over time in individuals with and without at least 10% of sites with PD ≥6mm.
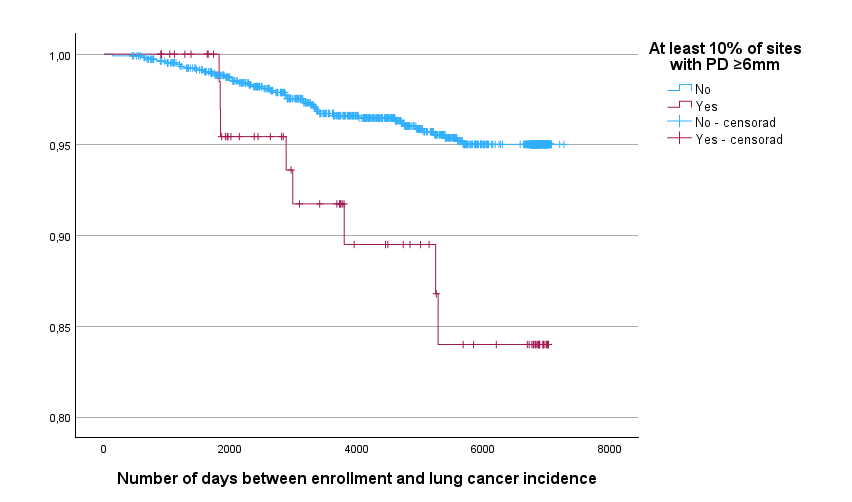


**Supplementary Figure S5.** Lung cancer incidence over time in individuals with and without at least 10% of sites with CAL ≥3mm.
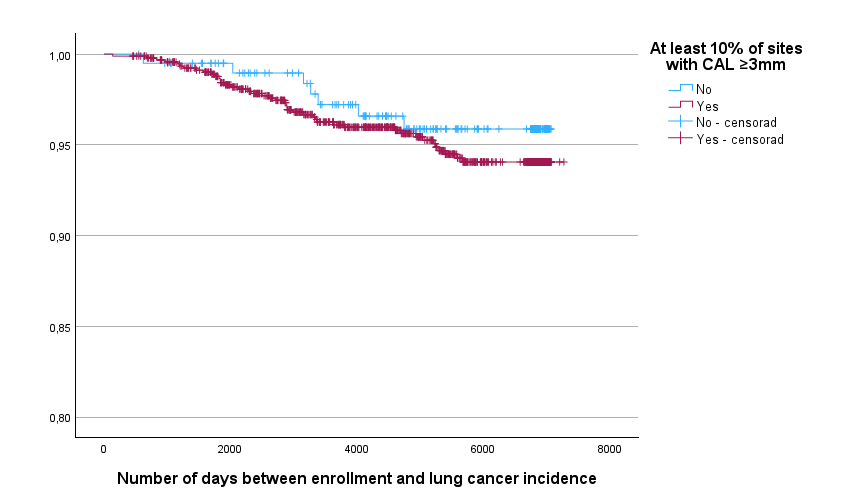


**Supplementary Figure S6.** Lung cancer incidence over time in individuals with and without at least 20% of sites with CAL ≥3mm.
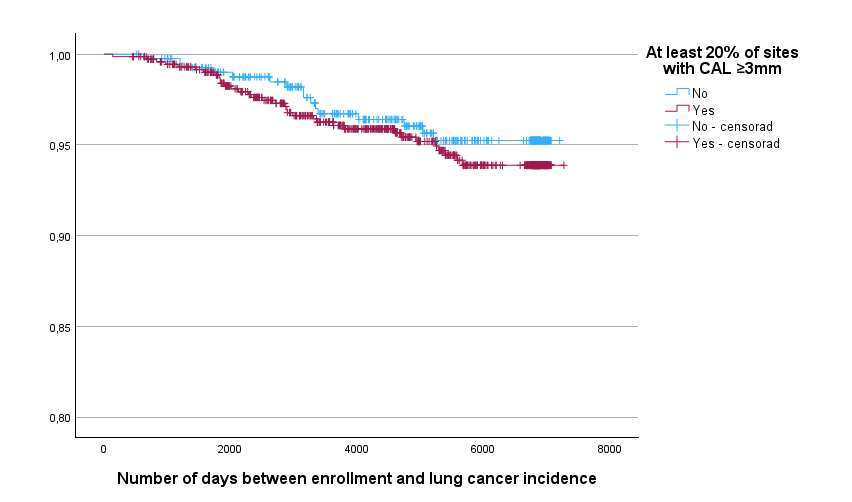


**Supplementary Figure S7.** Lung cancer incidence over time in individuals with and without at least 30% of sites with CAL ≥3mm.
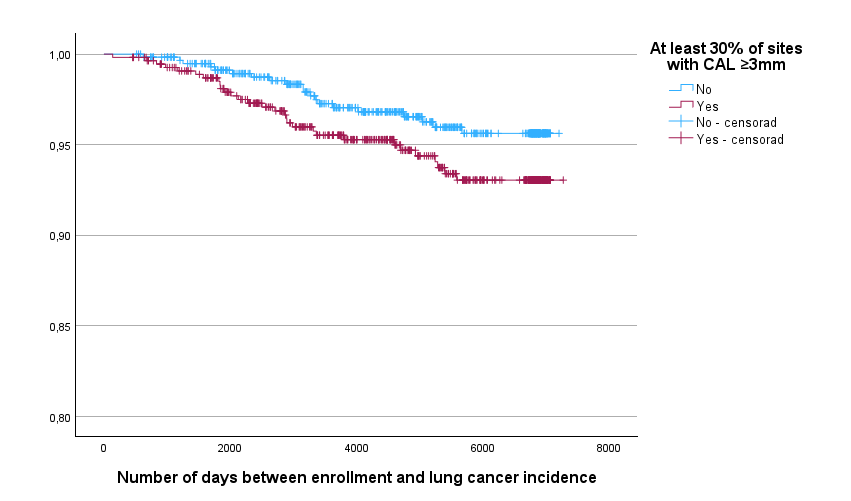


**Supplementary Figure S8.** Lung cancer mortality over time in individuals with and without severe tooth loss.
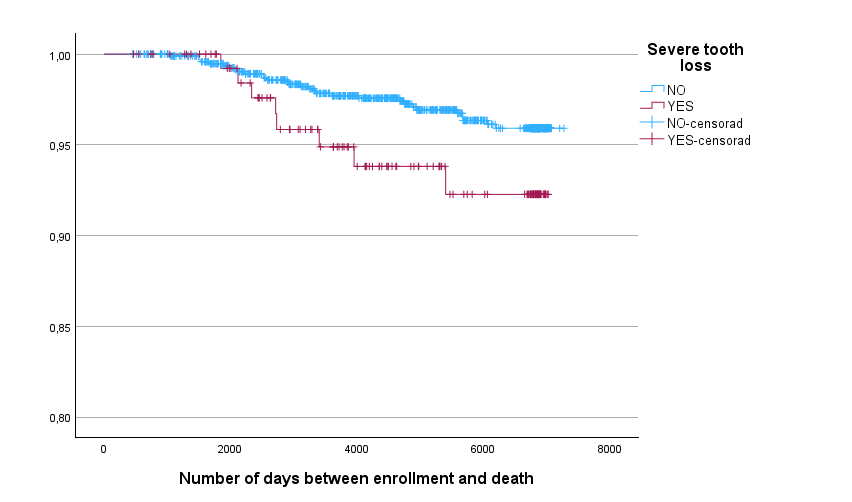


**Supplementary Figure S9.** Lung cancer mortality over time in individuals with and without at least 10% of sites with PD ≥4mm.
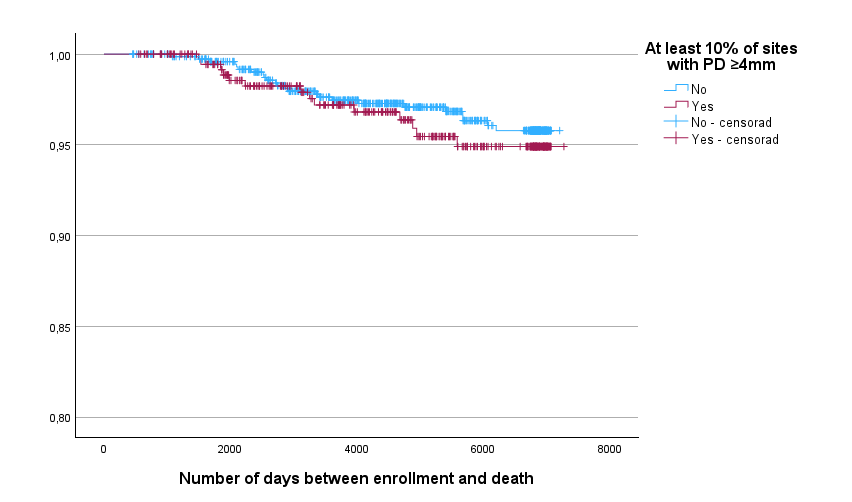


**Supplementary Figure S10.** Lung cancer mortality over time in individuals with and without at least 10% of sites with PD ≥5mm.
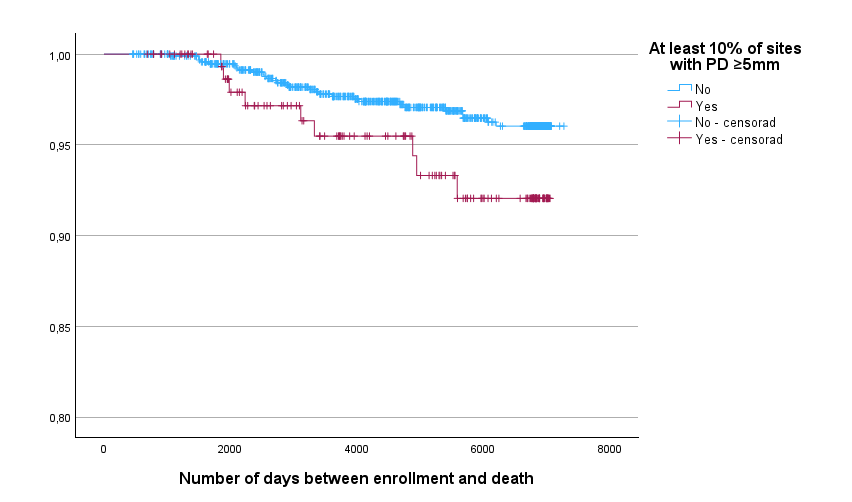


**Supplementary Figure S11.** Lung cancer mortality over time in individuals with and without at least 10% of sites with PD ≥6mm.
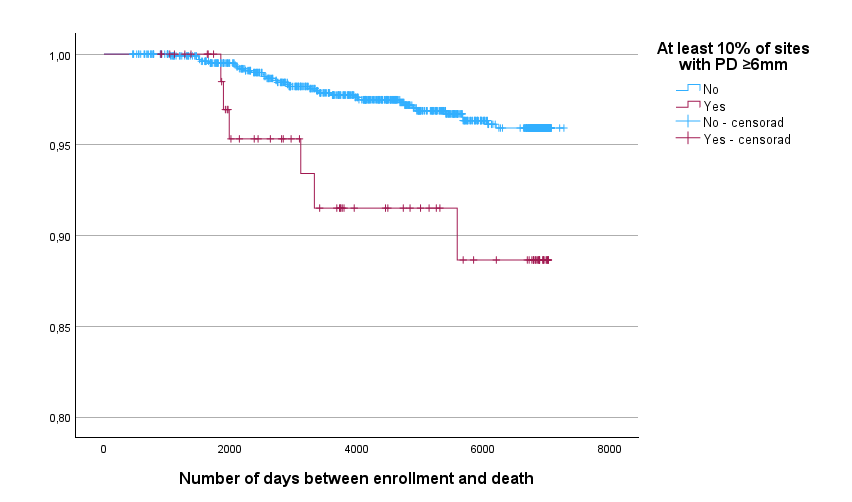


**Supplementary Figure S12.** Lung cancer mortality over time in individuals with and without at least 10% of sites with CAL ≥3mm.
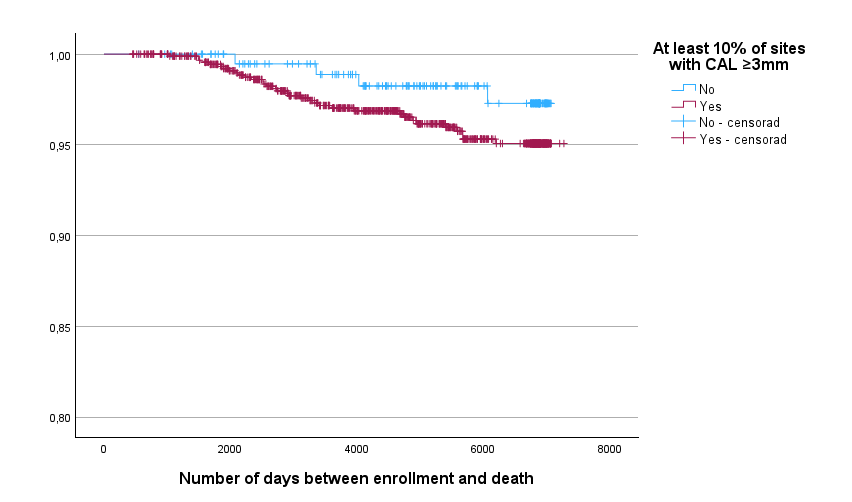


**Supplementary Figure S13.** Lung cancer mortality over time in individuals with and without at least 20% of sites with CAL ≥3mm.
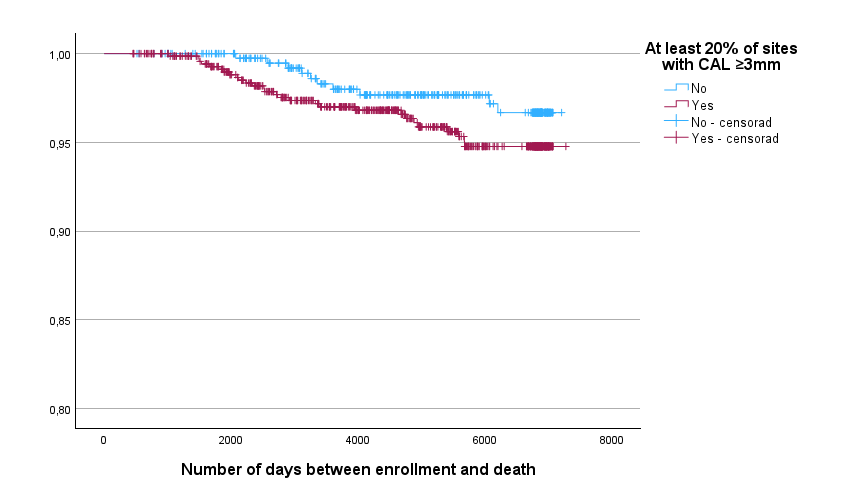


**Supplementary Figure S14.** Lung cancer mortality over time in individuals with and without at least 30% of sites with CAL ≥3mm.
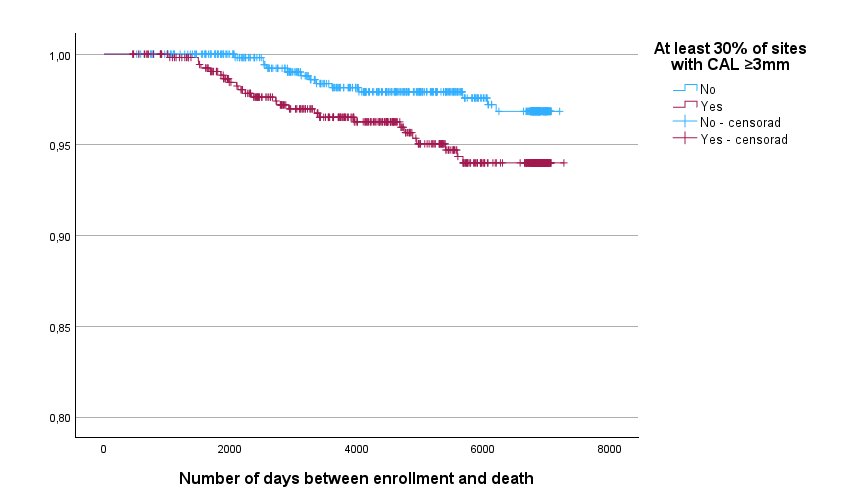


**Supplementary figure 15. Schoenfeld residuals for tooth loss and lung cancer incidence (fully adjusted model).**


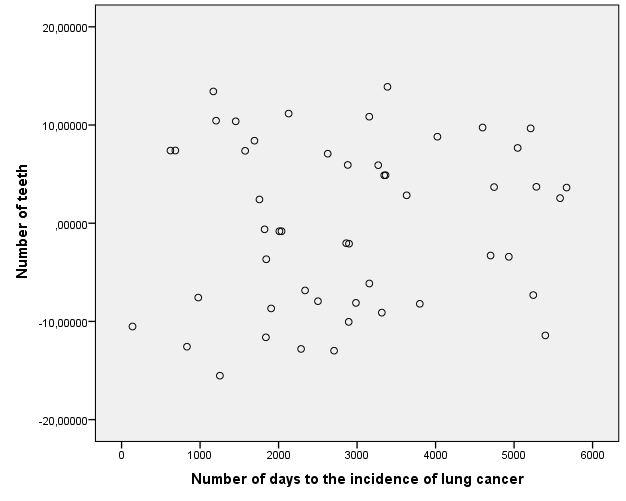


**Supplementary figure 16. Schoenfeld residuals for Mean PD and lung cancer incidence (fully adjusted model).**


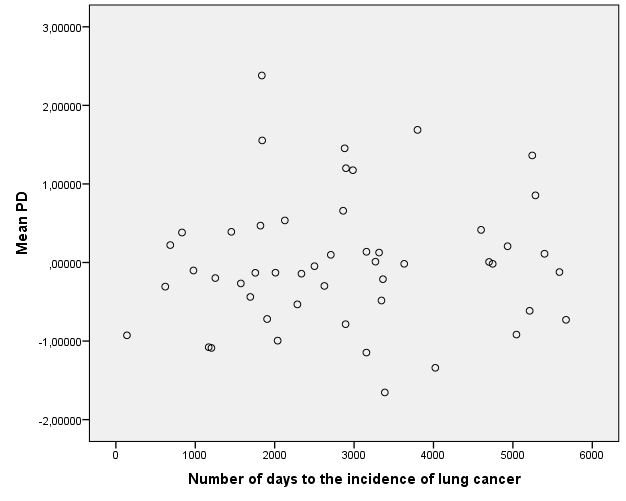


**Supplementary figure 17. Schoenfeld residuals for Mean AL and lung cancer incidence (fully adjusted model).**


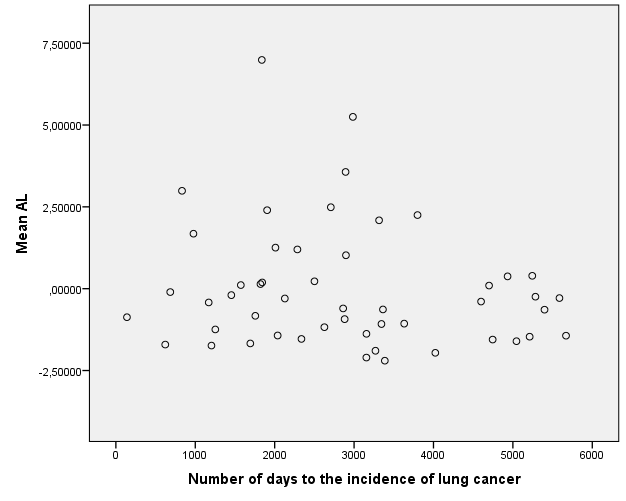


**Supplementary figure 18. Schoenfeld residuals for tooth loss and lung cancer death (fully adjusted model).**


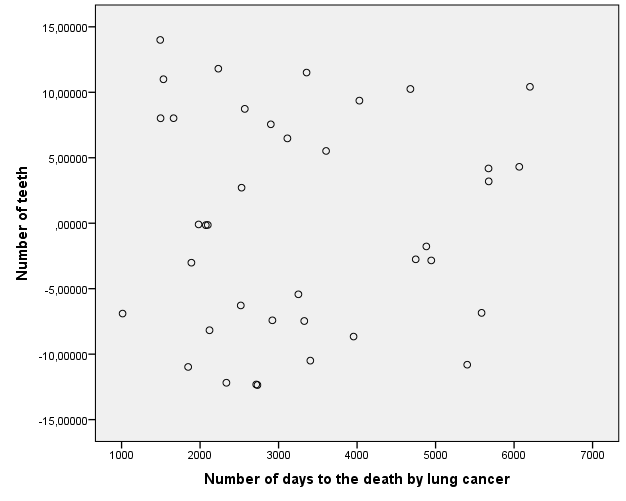


**Supplementary figure 19. Schoenfeld residuals for Mean PD and lung cancer death (fully adjusted model).**


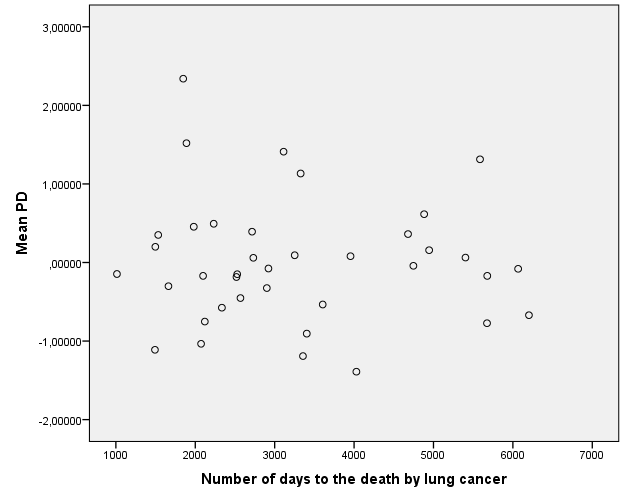


**Supplementary figure 20. Schoenfeld residuals for Mean AL and lung cancer death (fully adjusted model).**


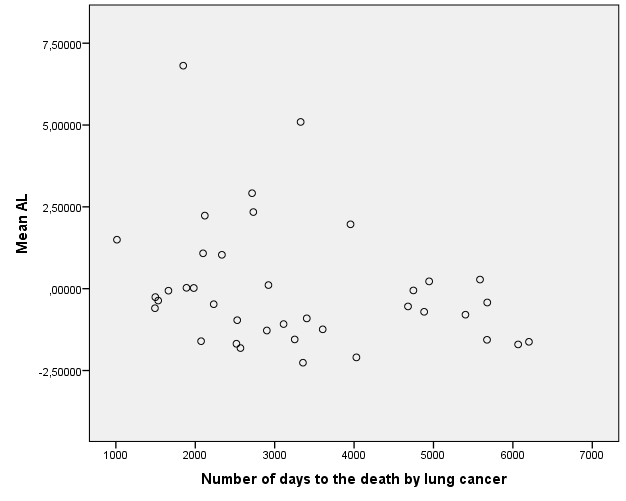


**Supplementary figure S21** - Cumulative incidence of lung cancer according to the presence of severe tooth loss.


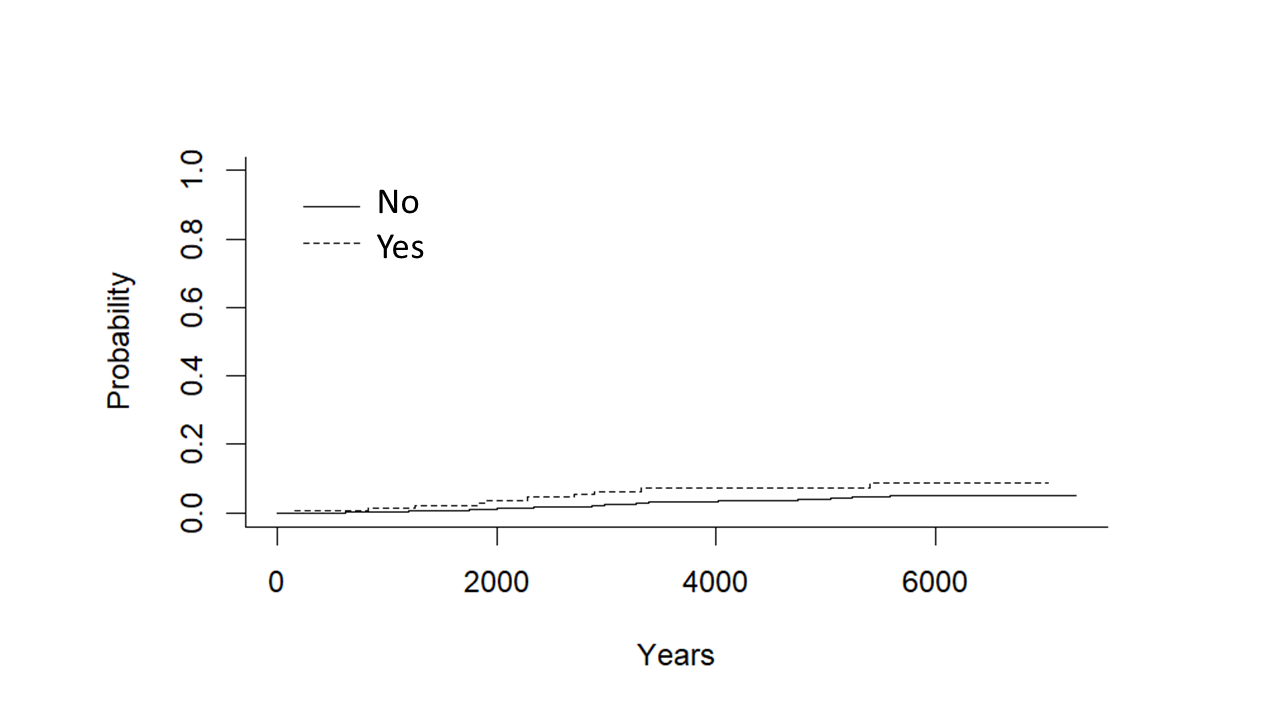


**Supplementary figure S22** - Cumulative incidence of lung cancer by probing depth thresholds (≥10% of sites with PD ≥4 mm, PD ≥5 mm, PD ≥6 mm).


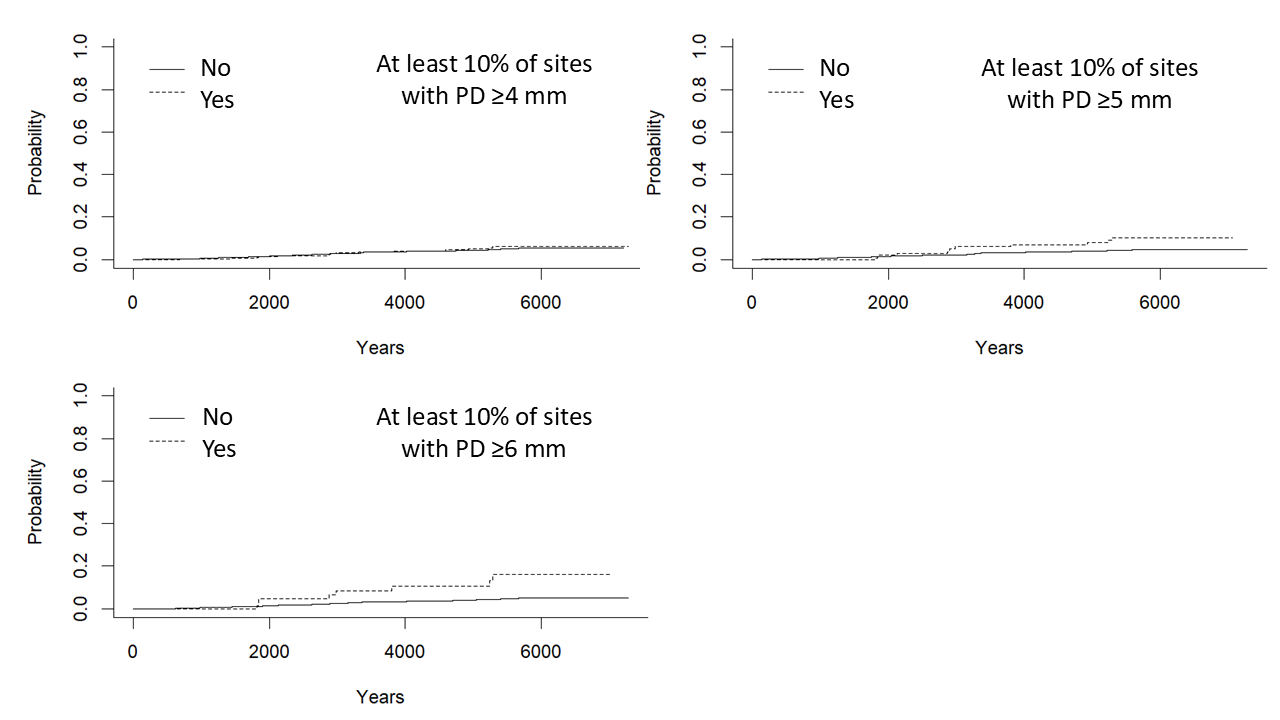


**Supplementary figure 23** - Cumulative incidence of lung cancer by clinical attachment loss thresholds (≥10%, ≥20%, ≥30% of sites with CAL ≥3 mm).
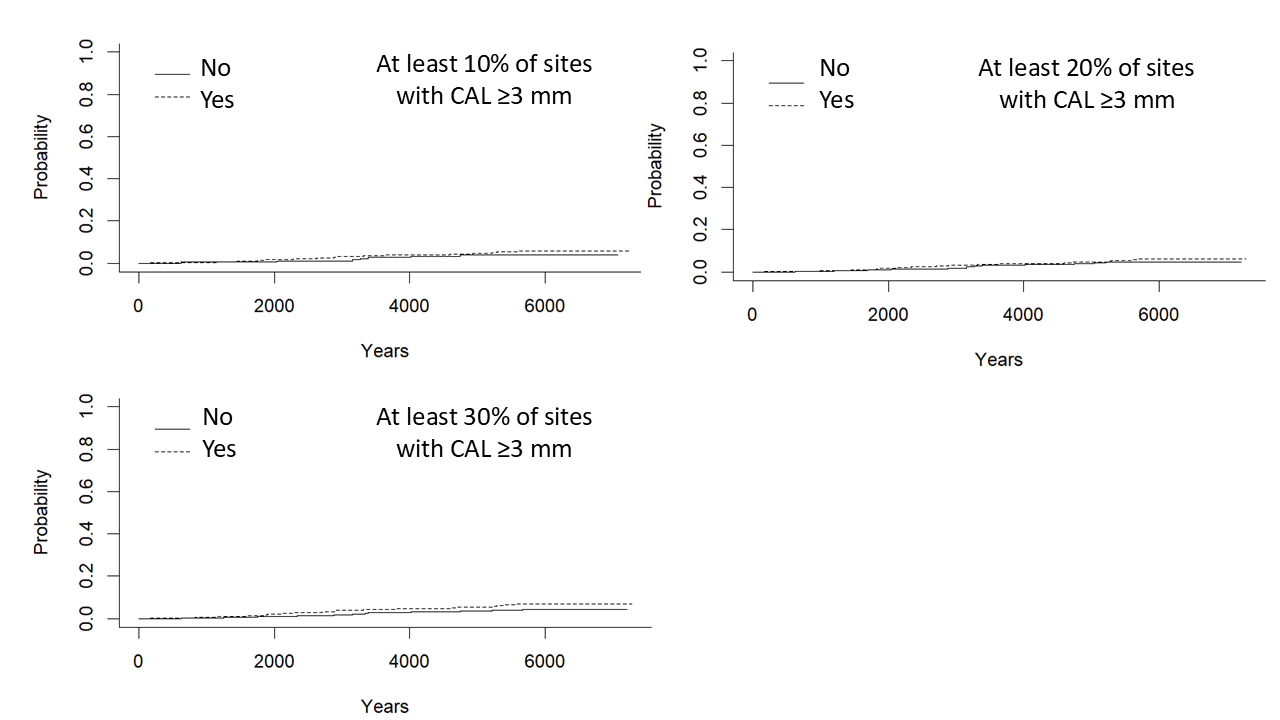

Supplement: Supplementary file 1 — Table S1: Frequency distribution for the older adults included and excluded in the periodontal sub‐study, along with comparison between these individuals. Excluded participants were those who were edentulous, systemically unwell, or not enrolled in the dental and periodontal examination. Table S2: Multivariate Cox regression analysis for lung cancer outcomes excluding participants who developed lung cancer within the first 2 years of follow‐up (n = 2). Table S3: Multivariate Cox regression analysis for lung cancer outcomes excluding participants who developed lung cancer within the first 2 years of follow‐up (n = 2). Table S4: Univariate Cox regression analysis for lung cancer outcomes. Table S5: Multivariate Cox regression analysis for lung cancer outcomes. Table S6: Multivariate cox regression analysis for lung cancer outcomes stratified by smoking status. Table S7: Fine–Gray subdistribution hazard models accounting for non–lung‐cancer death as a competing event. Figure S1: Lung cancer incidence over time in individuals with and without severe tooth loss. Figure S2: Lung cancer incidence over time in individuals with and without at least 10% of sites with PD ≥ 4 mm. Figure S3: Lung cancer incidence over time in individuals with and without at least 10% of sites with PD ≥ 5 mm. Figure S4: Lung cancer incidence over time in individuals with and without at least 10% of sites with PD ≥ 6 mm. Figure S5: Lung cancer incidence over time in individuals with and without at least 10% of sites with CAL ≥ 3 mm. Figure S6: Lung cancer incidence over time in individuals with and without at least 20% of sites with CAL ≥ 3 mm. Figure S7: Lung cancer incidence over time in individuals with and without at least 30% of sites with CAL ≥ 3 mm. Figure S8: Lung cancer mortality over time in individuals with and without severe tooth loss. Figure S9: Lung cancer mortality over time in individuals with and without at least 10% of sites with PD ≥ 4 mm. Figure S10: Lung cancer mortality over [file ODI-32-1460-s001.docx]
